# Supplementary material for: A Tri-Component (Glomerular, Tubular, and Metabolic) Assessment of Renal Function in Acute Heart Failure
Source: J Clin Med. 2024 Dec 20;13(24):7796. doi: 10.3390/jcm13247796 (PMC11727872; doi:10.3390/jcm13247796)
Supplement: Supplementary file 1 [file jcm-13-07796-s001.zip › jcm-3340989-supplementary.pdf]

Supplementary Material Table S1. Prevalence of renal dysfunction by the number of renal dysfunctions.

**Profile of renal dysfunction (number of renal dysfunctions)**	**no renal dysfunction**	**mono-component renal dysfunction**	**bi-component renal dysfunction**	**tri-component renal dysfunction**
Number of patients	86	106	41	10
Glomerular dysfunction<br>*eGFR on admission < 60 ml/min/1.73m²*	0 (0%)	30 (28%)	28 (68%)	10 (100%)
Tubular dysfunction<br>*spot urine sodium on day-1, ⩽ 60 mmol/l*	0 (0%)	56 (53%)	27 (66%)	10 (100%)
Renal metabolic dysfunction<br>*HCO_{3^-} on admission <21 mmol/l*	0 (0%)	20 (19%)	27 (66%)	10 (10%)

eGFR—estimated glomerular filtration rate.

Supplementary Material Table S2. Clinical and laboratory determinants of creatinine serum level on admission.

	**Multivariate regression model; b-coefficient**	**p-value**
**Creatinine >1.2 mg/dl**		
R-value of the model= 0., p<0.0		
Age, years	0.382	<0.0001
Gender, male	0.225	0.007
Serum Na^+ , mmol/l	0.040	0.66
Systolic blood pressure, mmHg	0.036	0.69
Left ventricle ejection fraction, %	-0.014	0.88
NTproBNP, pg/ml	0.184	0.03
Bilirubin, mg/dl	0.023	0.79
Lactate, mmol/l	0.03	0.73
Aldosterone at day-1, ng/dl	0.268	0.003

NT-proBNP - N-terminal pro–B-type natriuretic peptide;.

Supplementary Material Table S3. Associations between covariates used in the multivariable model and prespecified endpoints.

Variable	HR (95% CI)	p
**1-year mortality risk**		
Age, years, per 1 year	0.998 (0.979-1.017)	0.831
Sex, male	1.23 (0.69-2.19)	0.476
Systolic blood pressure, mmHg, per 1 mmHg	0.989 (0.980-0.999)	0.021
Left ventricle ejection fraction, %, per 1 %	0.989 (0.968-1.101)	0.331
Haemoglobin, g/dl, per 1g/dl	0.93 (0.82-1.06)	0.271
Troponin I, ng/ml, per 1 ng/ml	1.03 (0.98-1.09)	0.334
Serum Na^+ ,mmol/l, per 1mmol/l	0.912 (0.869-0.958)	0.002
NTproBNP, pg/ml, per 1 pq/ml	1.00004 (1.00002-1.00007)	0.0002
**1-year mortality or heart failure rehospitalization (whichever occurred first)**		
Age, years, per 1 year	0.999 (0.983-1.015)	0.904
Sex, male	1.09 (0.68-1.72)	0.726

Systolic blood pressure, mmHg, per 1 mmHg	0.987 (0.979-0.995)	0.001
Left ventricle ejection fraction, %, per 1 %	0.986 (0.969-1.004)	0.135
Haemoglobin, g/dl, per 1g/dl	0.93 (0.84-1.03)	0.183
Troponin I, ng/ml, per 1 ng/ml	1.01 (0.95-1.07)	0.745
Serum Na^+ ,mmol/l, per 1mmol/l	0.918 (0.878-0.959)	0.0001
NTproBNP, pg/ml, per 1 pq/ml	1.00002 (1.000004-1.00005)	0.017

NTproBNP – N-terminal pro-B-type natriuretic peptide

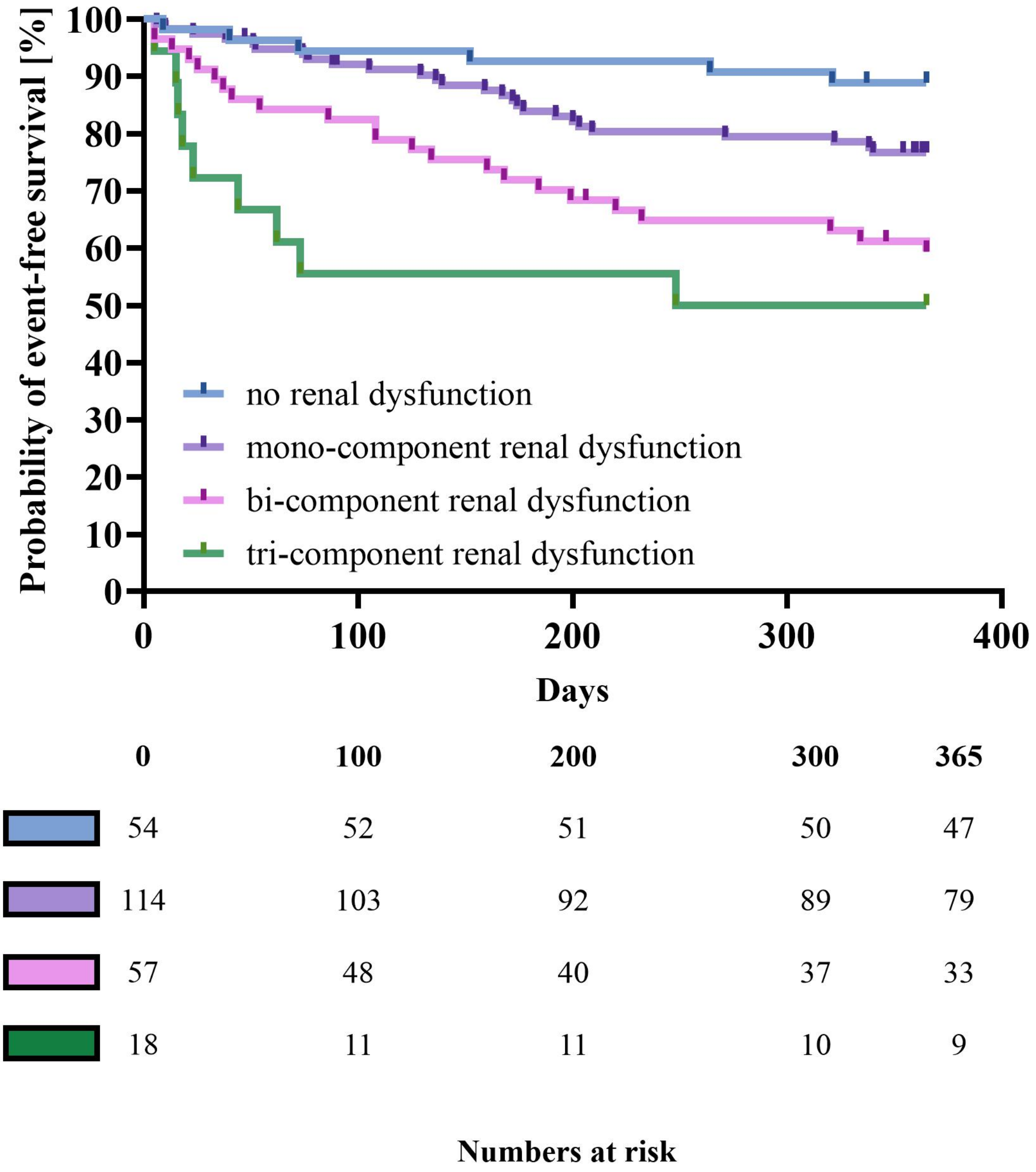

Supplementary material Figure S1. Kaplan-Meier curves for one year mortality by the renal dysfunction score. Log-rank, $p < 0.0001$. **(**Serum creatinine levels on admission > 1.2 mg/dL were used as a criterion, replacing the eGFR < 60 mL/min/1.73 m²**)**

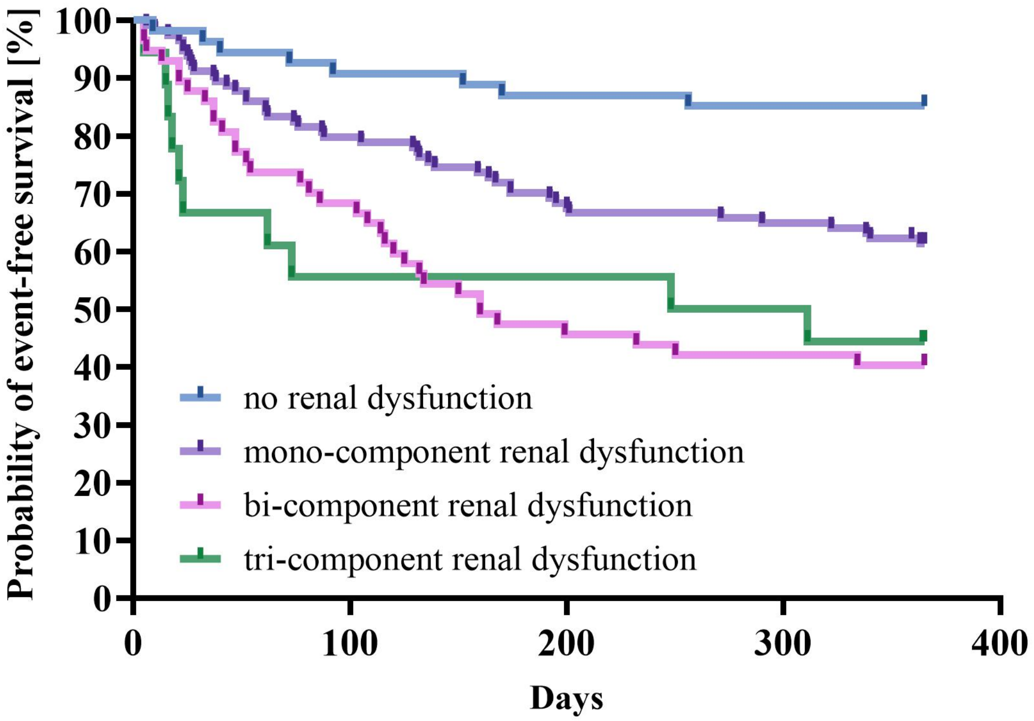

	0	100	200	300	365
	54	50	48	47	46
	114	92	78	75	68
	57	40	27	25	23
	18	11	11	10	7

Numbers at risk

Supplementary material Figure S2. Kaplan-Meier curves for death or heart failure rehospitalization (whichever occurred first) by the renal dysfunction score. Log-rank, $p<0.0001$. **(**Serum creatinine levels on admission > 1.2 mg/dL were used as a criterion, replacing the eGFR < 60 mL/min/1.73 m^2**)**
